# The Study of Derivatization Prior MALDI MSI Analysis—Charge Tagging Based on the Cholesterol and Betaine Aldehyde

**DOI:** 10.3390/molecules26092737

**Published:** 2021-05-06

**Authors:** Przemyslaw Mielczarek, Tymoteusz Slowik, Jolanta Helena Kotlinska, Piotr Suder, Anna Bodzon-Kulakowska

**Affiliations:** 1 Laboratory of Proteomics and Mass Spectrometry, Maj Institute of Pharmacology, Polish Academy of Sciences, Smetna 12 St., 31-343 Krakow, Poland; mielczar@if-pan.krakow.pl; 2 Experimental Medicine Center, Medical University of Lublin, Jaczewskiego 8D St., 20-090 Lublin, Poland; tymoteuszslowik@umlub.pl; 3 Department of Pharmacology and Pharmacodynamics, Medical University of Lublin, Chodzki 4a St., 20-093 Lublin, Poland; jolanta.kotlinska@umlub.pl; 4 Department of Analytical Chemistry and Biochemistry, Faculty of Materials Science and Ceramics, AGH University of Science and Technology, A. Mickiewicza 30 Ave., 30-059 Krakow, Poland; piotr.suder@agh.edu.pl

**Keywords:** cholesterol, betaine aldehyde, mass spectrometry (MS), mass spectrometry imaging (MSI)

## Abstract

Mass spectrometry imaging is a powerful tool for analyzing the different kinds of molecules in tissue sections, but some substances cannot be measured easily, due to their physicochemical properties. In such cases, chemical derivatization could be applied to introduce the charge into the molecule and facilitate its detection. Here, we study cholesterol derivatization with betaine aldehyde from tissue slices and evaluate how different sample preparation methods influence the signal from the derivatization product. In this study, we have tested different solutions for betaine aldehyde, different approaches to betaine aldehyde deposition (number of layers, deposition nozzle height), and different MALDI matrices for its analysis. As a result, we proved that the proposed approach could be used for the analysis of cholesterol in different tissues.

## 1. Introduction

About 25% of the whole-body cholesterol is deposited in the brain. Of this, 70% builds up in the myelin shed of oligodendrocytes around the axons, whereas 30% is localized in the plasma membranes of neurons and astrocytes [1]. Because the lipoproteins cannot pass through the brain–blood barrier (BBB), *de novo* synthesis of cholesterol is the only source of this molecule in the brain. The astrocytes are the main channel for cholesterol delivery, as they produce two to three times more of this molecule than the neurons. They also produce apolipoprotein E (apoE), which is a major transport protein for extracellular cholesterol. Neurons are able to produce enough cholesterol to survive and grow, but additional cholesterol is necessary for the formation of mature synapses [2]. Cholesterol is responsible for the shape of the membranes and signal transduction. Its depletion in neurons impairs synaptic vesicle exocytosis, neuronal activity, and neurotransmission, leading to dendritic spine and synapse degeneration [3]. Moreover, the demand for cholesterol changes throughout life and is the most intense during postnatal development [4]. There is growing evidence that alternations in cholesterol homeostasis are connected with CNS pathologies such as Alzheimer’s disease, Niemann–Pick disease, Rett syndrome [5], or Smith–Lemli–Opitz syndrome (SLOS) [6].

Cholesterol analysis using MALDI MS can be demanding because cholesterol is characterized by low proton affinity and low acidity. Nevertheless, several approaches have been proposed to make this analysis possible. Dufresne et al. and Xu et al. used silver sputtering to efficiently detect cholesterol as a silver adduct [cholesterol + ^109^Ag]^+^ [7,8]. Some of the approaches utilize salt doping and metal sputtering. For example, Dufrense et al. used sodium acetate and carbonate buffer mixture at pH 10.3 and sputtered a layer of gold (Au-CBS) that allowed for sodiated cholesterol observation [9].

Patti et al. used a direct silver coating of the NIMS surface (nanostructure-initiator MS specially prepared silicon wafers), with aqueous AgNO_3_, before tissue deposition, and laser desorption ionization (LDI) for cholesterol analysis [6]. A valuable review of different methods of cholesterol analysis could be found in [10].

For the compounds that do not ionize well, chemical derivatization is usually applied. Derivatization may also be used to increase the detection sensitivity and to decrease the limit of detection (LOD) or quantification (LOQ) of the compound. This process aims to increase the proton affinity of the analyte, or to introduce the charge for the analyte to increase the sensitivity of its analysis. Derivatization should be as specific as possible, occurring in solvents compatible with the MALDI ion source, and in conditions mild enough to avoid delocalization or tissue distortion. In some cases, the reaction product is designed to reach a maximal absorbance at 355 nm; thus, it could be analyzed without the MALDI matrix [11,12].

Derivatization requires a deposit of a layer of the chosen chemical compound over the surface of the analyzed tissue to allow the chemical reaction to occur. In our experiments, the SunCollect System was used for the matrix deposition before the MALDI MSI analysis. This approach is recognized as the “wet interface matrix deposition method”, using an optimized spray generator to produce extremely small matrix droplets. This feature enables its usage during the derivatization process.

**Figure 1:**
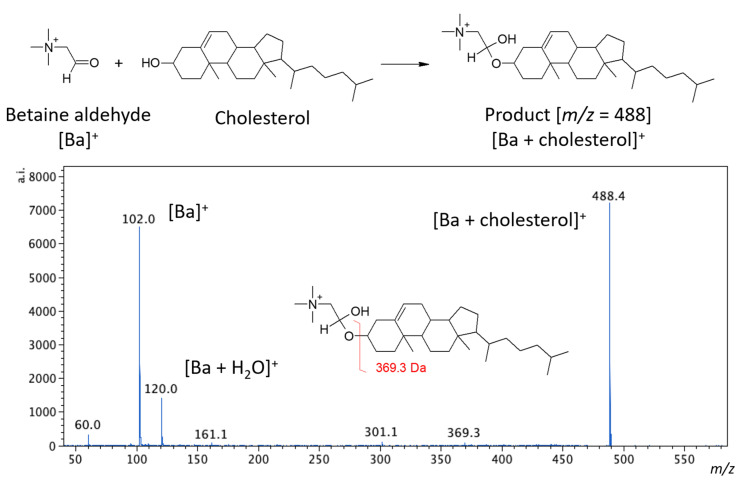
The scheme of cholesterol derivatization reaction with the betaine aldehyde (**top**) and the MS/MS spectrum for the derivatization product, obtained from the tissue (**bottom**).

Betaine aldehyde reacts selectively with alcohols. The reaction occurs between the aldehyde group of betaine and the hydroxyl group of the target alcohol (see Figure 1). In this reaction, hemiacetal salt is formed, and the obtained product is labeled with a charge. This could significantly improve the detection sensitivity [13]. This approach was proposed by Wu et al. [13] for reactive DESI and has been used previously by us for vascular graft analysis [14]. In this article, we intended to evaluate whether such derivatization is also possible for the MALDI analysis.

## 2. Results and Discussion

### 2.1. Matrix, Betaine Aldehyde and the Number of Layers on the Tissue Cross-Sections

**Figure 2:**
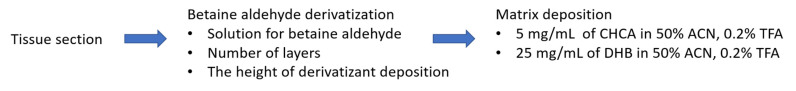
The scheme of sample preparation before MSI analysis with tested alternations.

The general scheme of the substance derivatization in the MSI experiment comprised of depositing the derivative agent (in our case, betaine aldehyde) in an appropriate solution, followed by covering the tissue slice with the appropriate matrix (Figure 2).

In the first part of the experiment, two different solvents for betaine aldehyde were used: a saturated solution of betaine aldehyde in 100% ACN, and a 0.1% betaine aldehyde in an ACN/H_2_O solution (2:1, *v*/*v*). Additionally, the influence of the number of betaine aldehyde layers on the intensity of the signal from the product of derivatization was checked. In the last step, two matrices, the DHB (25 mg/mL in ACN/H_2_O, 1:1, *v*/*v* + 0.2% TFA) and the CHCA (5 mg/mL in ACN/H_2_O, 1:1, *v*/*v* + 0.2% TFA), were applied.

For all the comparisons, 10 spectra from the same most intensive points within the analyzed tissue were taken, and the average values of peak intensity and standard deviations were calculated for the ion from the derivatized cholesterol.

#### 2.1.1. Betaine Aldehyde Solution

**Figure 3:**
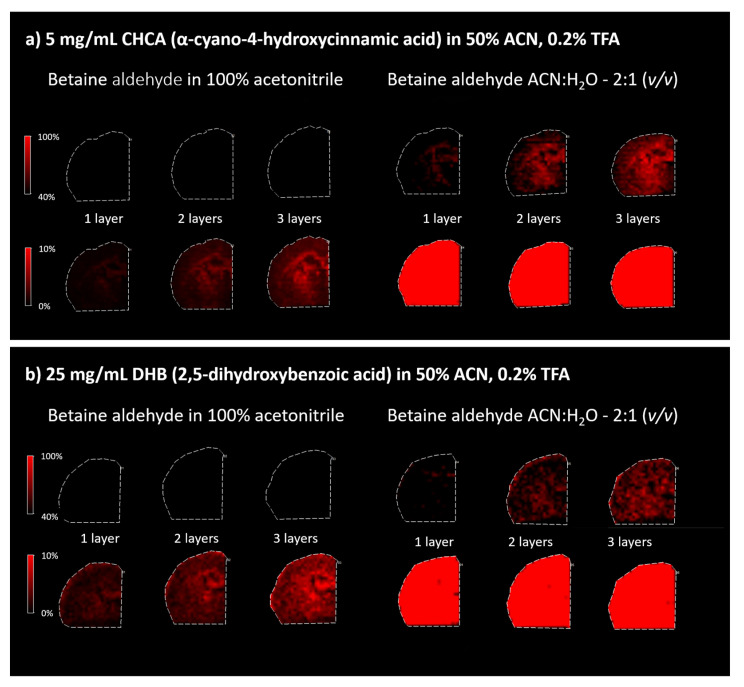
The results of the analysis for different matrices, betaine aldehyde solutions, and the number of layers. (**a**) CHCA and (**b**) DHB matrices. Left panels: saturated solution of betaine aldehyde in 100% acetonitrile. Right panels: solution of 0.1% betaine aldehyde in ACN:H_2_O (2:1, *v*/*v*). The rows for each matrix present the heatmap for the same analysis with the changes in the % of displayed intensity. The top rows in (**a**,**b**) represent the intensity scale from 40 to 100%, where 100% is for the most intensive cholesterol signal. The bottom rows represent the scale adjustment of the intensity from 0 to 10%, which allows for the observation of signals of lower intensity, which are invisible when the 40–100% range is used.

Because betaine aldehyde reacts with alcohols, these solvents cannot be used for its application. Aqueous solutions for mass spectrometry imaging may result in the loss of image resolution. On the other hand, the water addition may help extract the analyte of interest from the tissue. In our case, the application of the betaine aldehyde in 100% ACN resulted in an overall less intensive signal from the derivatization product (*m*/*z* = 488.7) (left panels in Figure 3). When betaine aldehyde was dissolved in acetonitrile with water (ACN:H_2_O; 2:1), an overall higher signal was obtained (right panels in Figure 3).

#### 2.1.2. The Number of Betaine Aldehyde Layers applied for Cholesterol Derivatization

**Figure 4:**
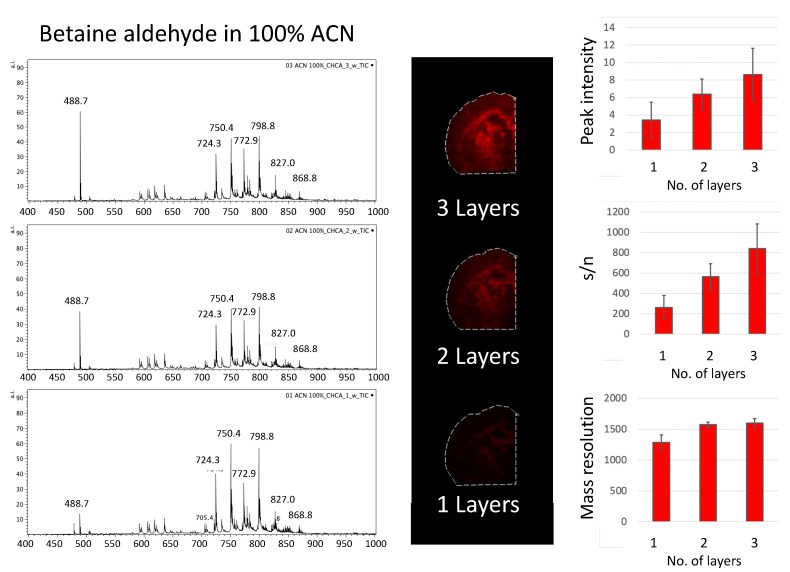
The influence of the number of layers of betaine aldehyde in 100% of acetonitrile on obtained mass spectra.

**Figure 5:**
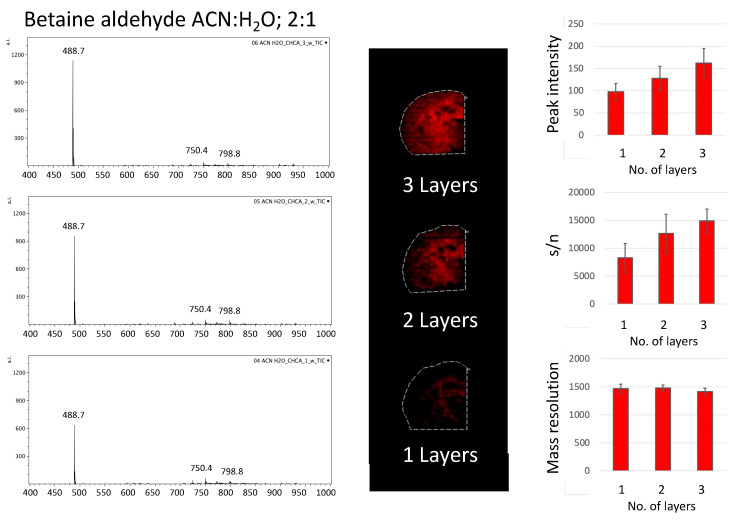
The influence of the number of layers of betaine aldehyde in acetonitrile with water solution, ACN:H_2_O = 2:1 (*v*/*v*), on obtained mass spectra.

Covering the area of the tissue slices with a polypropylene lid during betaine aldehyde solution deposition allowed us to test how different numbers of betaine aldehyde layers influenced the signal from the derivatization product. We decided to test one, two, and three layers of betaine aldehyde solution. The signal from the entire area of the tissue was taken. The intensity of the peak for cholesterol with betaine, along with its signal to noise ratio (s/n), and mass resolution were considered (Figure 4 and Figure 5).

Although the overall signal for derivatization with betaine aldehyde in 100% of acetonitrile is low, a clear linear dependence can be observed between the number of layers, the signal intensity, and the s/n ratio.

A similar effect could be observed for betaine aldehyde in an acetonitrile/water solution (ACN:H_2_O; 2:1, *v*/*v*), although a slight saturation of the signal’s response with the increasing number of layers could be observed when we considered the intensity from the whole tissue area (see Figure S3, Supplementary Materials). Additionally, in this case, the mass resolution for the monitored peak (*m*/*z* = 488.7) decreased somewhat with the number of layers.

When comparing different matrices for the analysis of derivativatized cholesterol on the tissue section, the CHCA matrix appeared to be better than DHB, as DHB-based spectra suffer from a lower resolution (see Figure 3).

### 2.2. The Height of the Nozzle for Betaine Aldehyde Spraying

The distance between the nozzle and the ITO surface is one of the most important parameters of the matrix for the deposition of other substances using the SunCollect System. Z = 1 mm is the highest position, and Z = 49 mm is the lowest position possible. Spraying the matrix from the topmost position results in a very dry spray, while the lowest position results in wetter vapor and better penetration of the solution into the tissue. We evaluated how the nozzle position influences the derivatization process and the resolution of the final heatmap of substance localization in the tissue.

**Figure 6:**
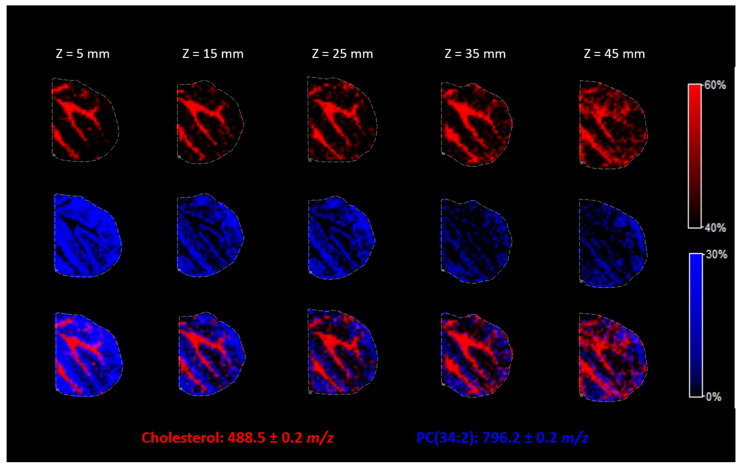
The influence of the nozzle height during matrix deposition on the spatial resolution of obtained results.

In Figure 6, we can see that the higher nozzle positions allowed for a better resolution of the picture. Thus, in the case of cholesterol derivatization, the highest possible nozzle position (Z = 5 mm) should be used to avoid lateral resolution loss.

### 2.3. Different Approaches for Sample Preparation before MSI Analysis

Two approaches to cholesterol derivatization with betaine aldehyde were experimentally tested. The first—the classical one—was based on spraying betaine aldehyde solution on the tissue (a mouse cerebellum cross-section) and standard CHCA matrix deposition prior to MSI. The second approach was based on preparing precoated ITO glasses with a derivatizing agent [15]. Briefly, ITO glass was coated with a CHCA matrix, applying the standard procedure (see section “Matrix deposition”). Afterward, the betaine aldehyde solution was sprayed onto the matrix layer using the same procedure that was used on the tissue cross-section (three layers in total). The 10 µm tissue slices were thaw-mounted directly onto the CHCA-precoated ITO glass. Another layer of the CHCA matrix was then sprayed onto the tissue according to the standard procedure. Additionally, one sample was prepared without cholesterol derivatization.

The preparation of precoated ITO glasses is usually used to save time during sample preparation, as classical matrix deposition on the day of the measurement is time-consuming [16]. Another advantage can be found for derivatization: the matrix and the derivative agent can be deposited on the ITO glass before the tissue cross-section thaw-mounting. This helps to avoid depositing the derivative agent on the tissue and reduces the risk of molecules delocalization during this process. It has to be stressed that in our case, another layer of CHCA matrix had to be applied on the tissue section to obtain the expected signal.

**Figure 7:**
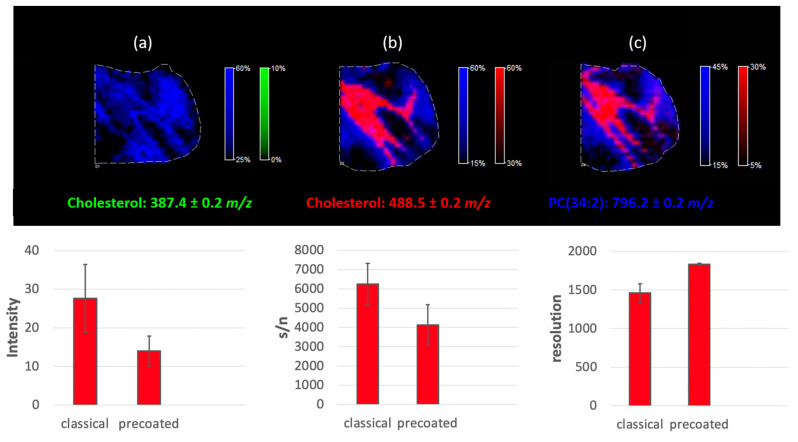
Different approaches to cholesterol analysis in a mouse cerebellum; (**a**) tissue covered only with a CHCA matrix, (**b**) tissue sprayed with a betaine aldehyde solution and covered with a CHCA matrix, (**c**) tissue mounted on the matrix, and betaine aldehyde precoated ITO glasses covered with CHCA matrix (please note the color scale bars and % of intensities).

As shown in Figure 7, cholesterol was visible for both approaches (uncoated and precoated glass), as an ion with *m/z* equals 488.5. PC (34:2) was used here as an endogenous standard and showed that standard lipid analysis is also possible with simultaneous cholesterol detection after each of the proposed strategies. The methodology involving the precoated ITO glasses resulted in a slightly higher spatial resolution of the final image. It is possible that avoiding tissue wetting is the cause. However, the signal from cholesterol is significantly weaker than the signal obtained after spraying the betaine aldehyde solution directly on the tissue. This is likely the result of avoiding the extraction process, which occurs while spraying the betaine aldehyde solution on the tissue. The reduction in ion intensities is more significant than the reduction in the s/n ratio. As a result, the mass resolution obtained for the tissue on a precoated ITO glass is much higher (see Figure 7). As we can see in Figure 7, it was impossible to detect any signal from naive cholesterol (the green color denotes a ratio of 387.4 *m*/*z*). The applications of only the CHCA matrix on the tissue (at 369.5 and 385.5 *m*/*z*) visible in SIMS were also checked with negative results. While betaine aldehyde retained a positive charge, we tested whether it was possible to obtain a signal from cholesterol after derivatization without CHCA matrix deposition, but for both approaches (the classical and the precoated), the experiments failed.

### 2.4. Cholesterol Analysis in the Different Tissues

**Figure 8:**
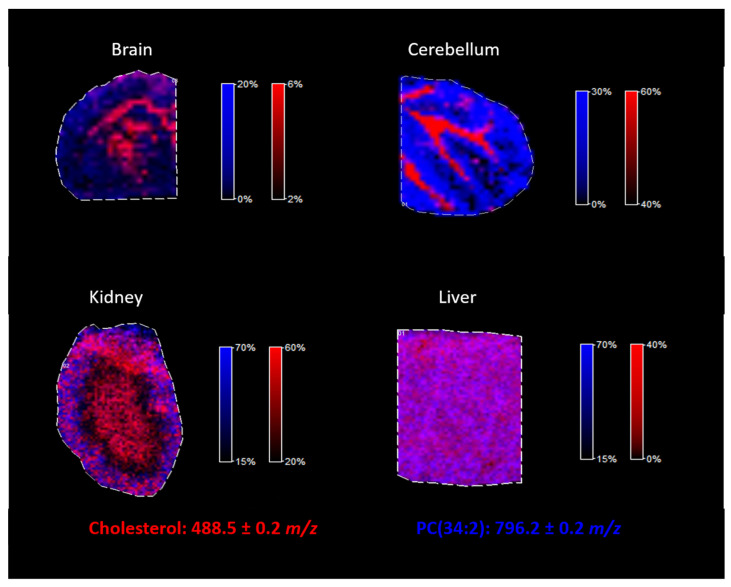
Cholesterol analysis in the different tissues after derivatization by betaine aldehyde with an optimized derivatization approach.

The proposed approach of cholesterol derivatization with betaine aldehyde was tested for different mouse tissues. As illustrated in Figure 8, by using the proposed methodology, cholesterol can be easily analyzed in tissues such as the brain, the cerebellum, the kidneys, and the liver, regardless of whether the tissue is heterogenous (such as the brain) or homogenous (such as the liver). Additionally, lipids analysis was still possible after the derivatization of cholesterol, as we demonstrated on an example of PC (34:2), identified in all analyzed mouse tissues.

## 3. Materials and Methods

### 3.1. Chemicals

During the experiments, the following chemicals were used: acetonitrile (ACN) and methanol (J.T. Baker, Deventer, The Netherlands), both at the HPLC-gradient grade. Betaine aldehyde, α-cyano-4-hydroxycinnamic acid (CHCA), 2,5-dihydroxybenzoic acid (DHB), trifluoroacetic acid (TFA) were at the highest available purity, received from Sigma–Aldrich/Merck (St. Louis, MO, USA) local distributor. The water used in the studies was purified using the Simplicity system from Millipore.

### 3.2. Animal Treatment

All experiments with animals were performed according to the respective Polish and European Communities Council Directives (86/609/EEC) and were approved by the local Ethics Committee (137/2018). The tissues from the mouse brain, rat kidney and liver were obtained as spared tissues from control animals used during other experiments, according to the so-called 3R (replacement/reduction/refinement) rule.

### 3.3. Tissue Sectioning

Immediately after isolation, until assayed, the tissues were kept in a laboratory freezer at −80 °C (NuAire NU9483, Plymouth, MN, USA). Approximately 1 h before cutting, the tissues were transferred to the cryomicrotome chamber (Cryotome FSE, Thermo Fisher Scientific, Chesire, UK). The temperature inside the chamber and the temperature of the specimen were set to −15 °C. Slices were cut and immediately thaw-mounted on the ITO glasses (Bruker-Daltonics, Bremen, Germany). The thickness of the tissue slices was 10 μm. The tissues were then dried under the vacuum for about 45 min. Prepared samples were stored at −80 °C in the hermetically sealed boxes until the analysis. Just prior to analysis, the samples were warmed up to an ambient temperature in a vacuum desiccator to prevent moisture condensation on the sample’s surface, and dried for about 1 h. Exemplary tissue sections were stained with cresyl violet. The procedure and the associated images are available in the supplementary materials (Figure S2, Supplementary Materials)

### 3.4. Matrix Deposition and Cholesterol Derivatization

Before each matrix deposition, an optical image (600 dpi) of the tissue cross-sections was registered for a glass slide with marked fiducials. Derivatization of cholesterol in the tissues was performed using a SunCollect device (SunChrom GmbH, Friedrichsdorf, Germany) by spraying a 0.1% betaine aldehyde solution in an ACN:H_2_O solution (2:1, *v*/*v*) with a constant flow rate equal to 60 µL/min, which yielded approximately 0.4 µg/cm^2^ of betaine aldehyde deposited on a single layer. For some experiments, a saturated betaine aldehyde solution in a pure ACN was used. A different number of layers was tested during experiments. The reaction between betaine aldehyde and cholesterol on the tissue sections occurs immediately, so it is not necessary to wait a specific time for the process to occur.

In the classical approach, matrix coating was performed after cholesterol derivatization. The tissue cross-sections were coated with a CHCA matrix solution (5 mg/mL in 1:1 *v*/*v* ACN:H_2_O + 0.2% TFA) or alternatively with a DHB matrix solution (25 mg/mL in 1:1 *v*/*v* ACN:H_2_O + 0.2% TFA) using a SunCollect device. This resulted in the deposition of approximately 2 μg of CHCA or 10 μg of DHB matrices per square millimeter of ITO glass slide. Briefly, each matrix was deposited on the tissue via the application of 10 layers with a line distance of 2 mm, a speed of 600 mm/min and with Z = 25 mm (nozzle height over the sample). The flow rate of the matrix solution was variable: 10 µL/min for the first layer, 20 µL/min for the second layer, 30 µL/min for the third layer, 40 µL/min for the fourth layer, and 60 µL/min for all other layers.

Covering individual tissue cross-sections on the ITO glass with a polystyrene lid allowed for testing and measuring different conditions on the same glass slide. It also allowed for a proper comparison of the obtained results (see Figure S1, Supplementary Materials). A polystyrene lid allowed for spraying different numbers of betaine aldehyde layers and protected against unwanted derivatization of adjacent tissue cross-sections (the derivatization is extremely sensitive). In terms of the matrix application, this enabled us to reach sharp matrix edging, which allowed for placing a larger number of tissue sections on a single ITO slide.

### 3.5. MALDI Measurements

Matrix-coated sections, after betaine aldehyde derivatization and without any derivatization, were subjected to imaging using a MALDI–TOF/TOF UltrafleXtreme MS (Bruker Daltonics, Bremen, Germany) with a Smartbeam II™ laser operating at 2 kHz. Ions were accelerated at 25 kV with a pulsed ion extraction of 120 ns and ion suppression up to 100 Da. Spectra were recorded in positive ion mode with reflectron, within an *m/z* range of 200–3000. They were externally calibrated with Peptide Calibration Standard II (Bruker Daltonics, Bremen, Germany) and a CHCA matrix ion [2M + H]^+^ with an *m/z* equal to 379.09. A raster width of 100 µm was applied to all samples, except for the imaging of mouse brain tissue, where the raster width was set to 200 µm. In total, 400 shots were collected from each ablation point with 20 shots at the raster spot, and the laser focus diameter was set to “4_large”. The FlexControl version 3.4 (Bruker Daltonics) was employed for spectra acquisition, and the FlexImaging version 4.0 was used for data processing and the creation of molecular images. All spectra were normalized against TIC. Mmass software (version 5.5.0, Open Source software developed by Martin Strohalm, Academy of Sciences, Prague, Czech Republic) was used for the spectra analysis [17]. To investigate the trend during measurement optimization, 10 spectra from the same most intensive points within the analyzed tissue were taken, and the average value of peak intensity and standard deviation were calculated for the ion from derivatized cholesterol.

## 4. Conclusions and Future Perspective

It was demonstrated in this study that cholesterol derivatization with betaine aldehyde could significantly improve cholesterol ionization during MALDI MSI. We have developed an optimal approach to reach a high spatial resolution and a low LOD. It was also shown that the proposed methodology allows for the simultaneous detection of cholesterol and lipids visible in positive ion mode, which was demonstrated for different mouse tissues. The optimal procedure of cholesterol derivatization in tissues relied on spraying a betaine aldehyde solution on the tissue from a height sufficiently low to avoid wetting the tissue, which significantly increased spatial resolution and avoided a loss of signal from the lipids. The choice of the solution for betaine aldehyde and the number of applied layers of this derivatization reagent depends on the intended purpose. Our study showed that with an ACN:H_2_O solution (2:1, *v*/*v*), we could observe a very strong signal, which is appropriate for measurements where the highest possible sensitivity is required. Conversely, a 100% ACN helped obtain a slightly lower signal, but allowed for an easier observation of different lipids simultaneously. Moreover, we had a better response with regard to the number of derivatization reagent layers. A CHCA appears to be better than the DHB matrix for cholesterol analysis. Alternatively, a methodology involving ITO glasses precoating with the derivatizing agent was developed to further increase spatial resolution during MSI, and to reduce the time between sample preparation and mass spectrometry measurement.

Because cholesterol plays an important role in different biochemical phenomena, we hope that this derivatization technique will be helpful in further research examining its significance.

## Data Availability

All the data present in the study are available upon request from the corresponding author.

## Supplementary Materials

Figure S1: (**a**) ITO glass slide partially covered with polystyrene lid during matrix deposition (semi-transparent lid visible on the right); (**b**) sharp edges of matrix obtaining after lid removal; (**c**) regions of ITO glass slide with different matrix and derivatizing strategy. Figure S2: Exemplary tissue sections stained with cresyl violet, with the heat-map of cholesterol distribution. Figure S3: A slight saturation of the signal’s response with the increasing number of layers could be observed when we consider the signal intensity from derivatized cholesterol from the whole tissue area.

## Sample Availability

Samples of the compounds are not available from the authors.
