# Supplementary material for: The Study of Derivatization Prior MALDI MSI Analysis—Charge Tagging Based on the Cholesterol and Betaine Aldehyde
Source: Molecules. 2021 May 6;26(9):2737. doi: 10.3390/molecules26092737 (PMC8124285; doi:10.3390/molecules26092737)
Supplement: Supplementary file 1 [file molecules-26-02737-s001.zip › molecules-1197635-supplementary.pdf]

The study of derivatization prior mass spectrometry imaging (MSI) analysis – charge tagging based on the cholesterol and betaine aldehyde

Przemyslaw Mielczarek [1], Tymoteusz Slowik [2], Jolanta Helena Kotlinska [3], Piotr Suder [4], Anna Bodzon-Kulakowska [4,*]

1 Laboratory of Proteomics and Mass Spectrometry, Maj Institute of Pharmacology, Polish Academy of Sciences, Smetna 12 st., 31-343 Krakow, Poland
2 Experimental Medicine Center, Medical University of Lublin, Jaczewskiego 8D st., 20-090 Lublin, Poland
3 Department of Pharmacology and Pharmacodynamics, Medical University of Lublin, Chodzki 4a st., Medical University, 20-093 Lublin, Poland
4 Department of Analytical Chemistry and Biochemistry, Faculty of Materials Science and Ceramics, AGH University of Science and Technology, A. Mickiewicza 30 ave., 30-059 Krakow, Poland
* Correspondence: abk@agh.edu.pl; Tel.: +48 12 6175083

Supplementary materials:

• Polystyrene lid – sample preparation

The way of dealing with different ways of sample preparation on a single glass during the optimization of the MSI tissue measurements:

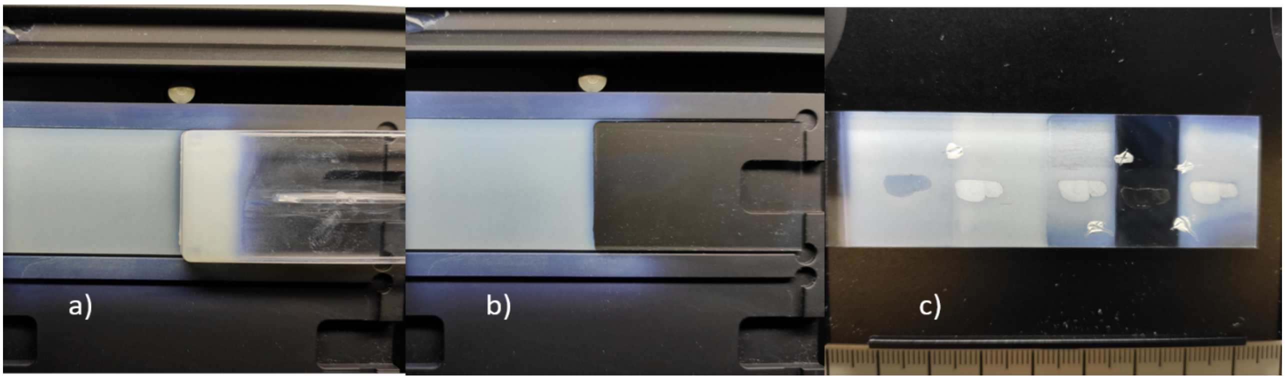

Figure S1. (a) ITO glass slide partially covered with polystyrene lid during matrix deposition (semi-transparent lid visible on the right); (b) sharp edges of matrix obtaining after lid removal; (c) regions of ITO glass slide with different matrix and derivatizing strategy.

• Nissl staining

Histological Nissl staining of the tissue sections after MALDI measurements were proceed as follows:

1. 5 minutes washes in 96% ethanol,
2. 5 minutes washes in 96% ethanol,
3. Drying for 5 min in desiccator,
4. Immersion in the water for 1 minute,
5. Cresyl violet stain* - 15 minutes
6. Dipping in water to remove cresyl violet solution (Sigma-Aldrich)
7. 30 second 50% ethanol
8. 30 second 95% ethanol
9. 30 second Histochoice Clearing Agent (Sigma-Aldrich)
10. Mounting tissue section with DPX mountant for histology (Sigma-Aldrich)
11. Obtaining a picture of tissue section

*Cresyl violet stain: 1.25g cresyl violet acetate and 0.75 ml glacial acetic acid to 250 ml warm H_20, cool and filter

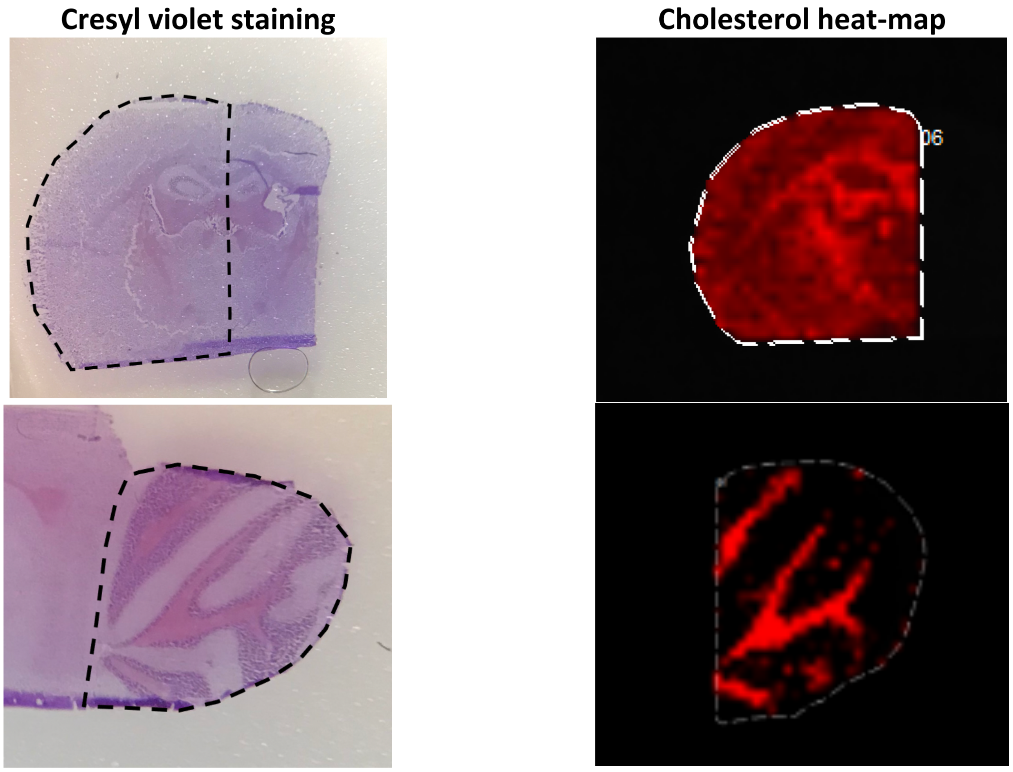

Figure S2. Exemplary tissue sections stained with cresyl violet, with the heat-map of cholesterol distribution.

- ## The number of betaine aldehyde layers – cholesterol derivatization

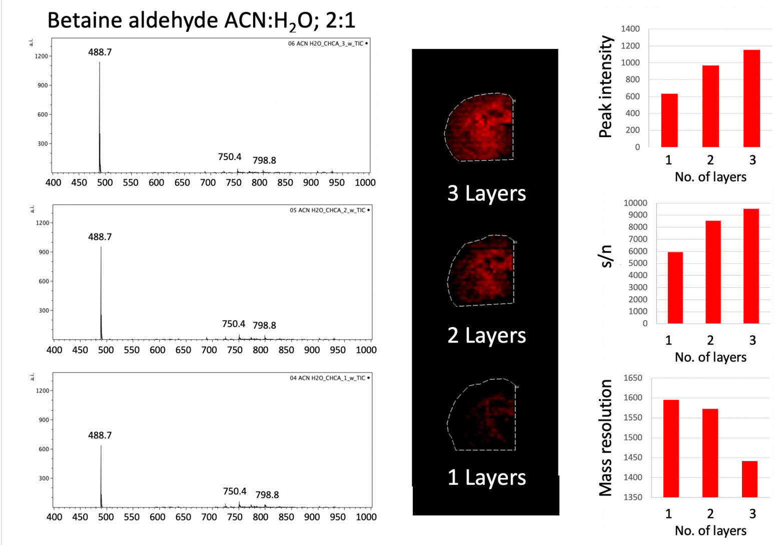

Figure S3. A slight saturation of the signal's response with the increasing number of layers could be observed when we consider the signal intensity from derivatized cholesterol from the whole tissue area.
